# Assessment of simple sequence repeats signature in hepatitis E virus (HEV) genomes

**DOI:** 10.1186/s43141-022-00365-w

**Published:** 2022-05-17

**Authors:** Md Gulam Jilani, Safdar Ali

**Affiliations:** Clinical and Applied Genomics (CAG) Laboratory, Department of Biological Sciences, Aliah University, IIA/27, Newtown, Kolkata, 700160 India

**Keywords:** Hepatitis E virus, Simple sequence repeats, Imperfect microsatellite extractor (IMEx), Incidence, Prevalence, Phylogenetic

## Abstract

**Background:**

Hepatitis E virus (HEV) is small (27–34 nm diameter) non-enveloped with positive sense ssRNA genome. Microsatellites or simple sequence repeats (SSR) are short tandem repeat sequences present across coding and non-coding regions of both prokaryotes and eukaryotes. They are involved with genome function and evolution at multiple levels.

**Results:**

The complete genome sequences of 22 HEV genomes of the family *Hepeviridae* and genus *Orthohepevirus* (21 species) and *Piscihepevirus* (1 species) were extracted from NCBI database (http://www.ncbi.nlm.nih.gov/). The extraction of microsatellites was done using Imperfect Microsatellite Extractor (IMEx) in ‘Advance-Mode’. The average genome size of the studied HEV genomes was 7003nt and it ranged from 6649nt (HEV11) to 7310nt (HEV22). The average GC content of the genomes was ~ 55%. A total of 519 SSRs and 21 cSSRS were extracted from the HEV genomes with an average incidence of 24 per genome ranging from 14 (HEV13) to 34 (HEV19). The cSSR incidence ranged from 0 (eight species) to 4 (HEV19). The genomes with no cSSR incidence had an SSR incidence range from 14 to 28. There were just four hexa-nucleotide repeat motifs and 5 penta-nucleotide repeat motifs observed. The most prevalent mono-, di-, and tri-nucleotide repeat motifs were “C”, “GT/TG”, and “GAC/CTG” respectively. The studied genomes had a minimum of ~ 90% incident SSRs present in the coding regions. Viruses with same or similar hosts are placed together on the phylogenetic tree implicating viral host being one of the driving forces for evolution. Conclusions

Host range in viruses is being decided by multiple factors aided by the unique genome SSR signature and genomes of varied compositions need to be analyzed to forge a widely acceptable rule for predicting the same.

**Supplementary Information:**

The online version contains supplementary material available at 10.1186/s43141-022-00365-w.

## Background

Hepatitis E virus is a small (27–34 nm diameter) non-enveloped positive sense, ssRNA virus. The size of the genome is 6.4–7.2 kb. Hepatitis, a very common disease around the world is generally caused by Hepatitis E (HEV) virus. Hepatitis E normally causes acute infection but the infection can change into chronic in immunodeficient people [1].

There are 8 different serotypes of HEV (serotype 1–8). On the basis of epidemiology, there are two conclusive HEV infections in human. HEV1 and HEV2 are predominantly found in developing countries and transmit from one person to another by the fecal-oral route through contaminated water. The property of spreading of HEV1 and HEV2 means that often sporadic cases can lead to infection in large area if sanitation conditions are poor. In developed countries, HEV3 and HEV4 proliferate between animals including pigs, wild boars, and deer and occasionally infect to human zoonotically [2]. Serotype of HEV5 and HEV6 has been identified only in wild boars whereas the camel is the host of HEV7 and HEV8 [3]. The incidence of sporadic HEV infections have increased in developed country [4]. According to Rein et al. 2012, HEV infected over 20 million people annually of which around 70,000 people died due to acute hepatic disease [5]. HEV demonstrated diverse physiological manifestations like a spectrum of neurological symptoms and infection, outgrowth of hepatic tissue, hematological disease, kidney diseases, acute pancreatitis, myocarditis, arthritis, and autoimmune thyroiditis [6]. HEV has been isolated from neuronal cell, human placenta, breast milk, and urine [7–10]. Thus, it is evident that a completing understanding of the HEV is required to combat the problems associated with it.

Microsatellites or simple sequence repeats (SSR) are short tandem repeat sequences and have been reported across coding and non-coding regions of both prokaryotes and eukaryotes. They are involved with genome function and evolution at multiple levels. Present study focusses on understanding the genomics of HEV through various aspects of microsatellites: incidence, prevalence, composition, and localization.

## Results

### Genome features

The average genome size of the studied HEV genomes was 7003nt and it ranged from 6649nt (HEV11) to 7310nt (HEV22). The average GC content of the genomes was ~ 55% with only one virus HEV22 belonging to genus *Piscihepevirus* having GC content of less than 50%. The various features of the studied genomes have been summarized in Supplementary file 1.

### Microsatellite incidence

A total of 519 SSRs and 21 cSSRS were extracted from the HEV genomes with an average incidence of 24 per genome ranging from 14 (HEV13) to 34 (HEV19). Interestingly, HEV19 representing *Moose hepatitis E virus* (HEV 19) had not only the highest SSR incidence but was also at the maximum level for several other aspects like GC content (58.5%); sRA of 4.8; sRD of 44.5; cSSR incidence of 4;cRA of 0.57; cRD of 16.3; and cSSR% of 23.5.

**Fig. 1:**
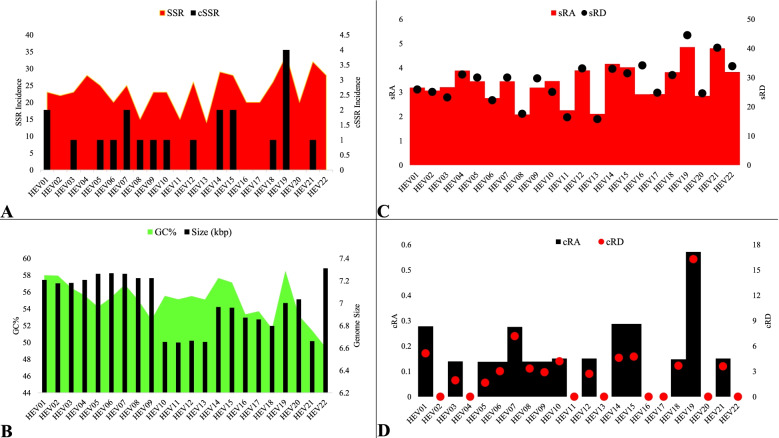
Genome features and microsatellite incidence. **A** Incidence of SSR and cSSR. **B** Size of genome and GC content. **C** RA and RD of SSR. **D** RA and RD cSSR

The genome size in relation to GC content and summary of incidence of SSRs and cSSRs along with RA and RD for SSR and cSSR have been represented in Fig. 1. Their details have been provided in Supplementary file 1. The cSSR incidence ranged from 0 (Eight species) to 4 (HEV19). The genomes with no cSSR incidence had an SSR incidence range from 14 to 28.

### Microsatellite composition

**Fig. 2:**
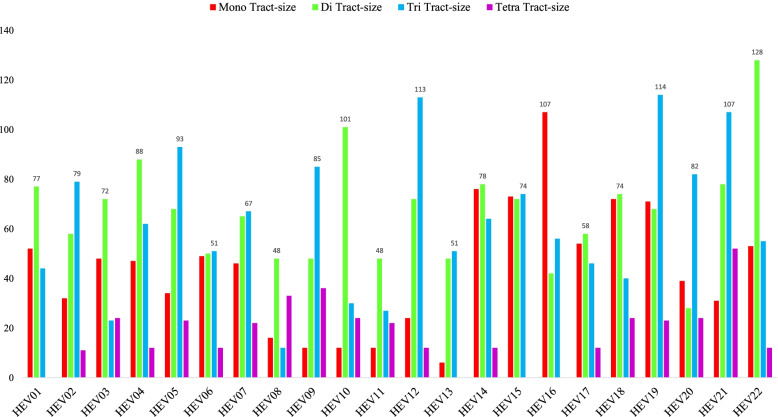
Total tract size of mono-, di- and tri- and tetra- nucleotides in Hepeviridae genomes

**Fig. 3:**
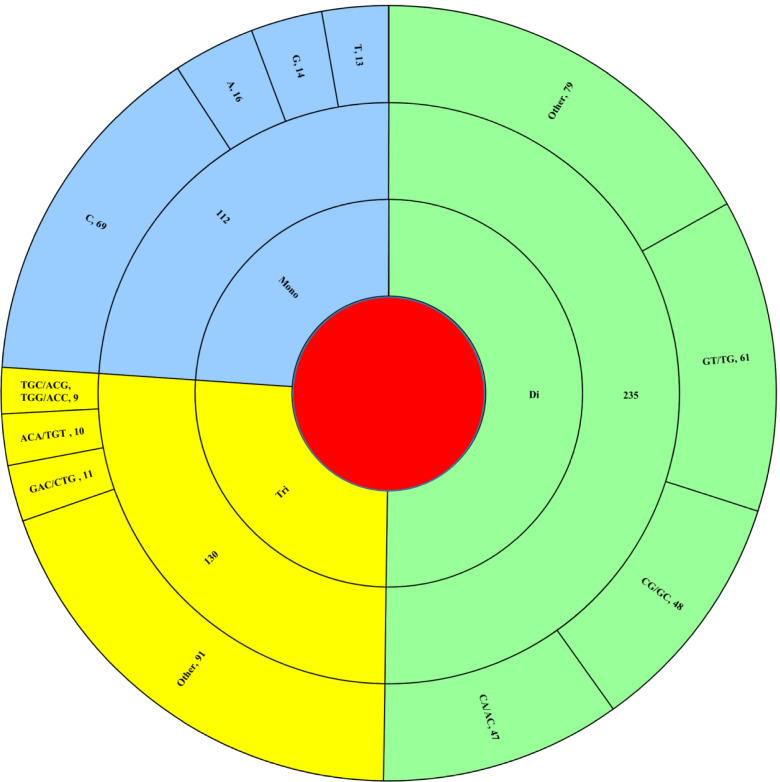
Motif composition of mono-, di-, and tri-nucleotide Hepeviridae genomes

The microsatellite composition is defined by two aspects namely tract size (number of repeats) and motif constitution (mono-, di-, tri). The diversity of microsatellites will have another aspect: sequence of the repeat motif. There were just four hexa-nucleotide repeat motifs and 5 penta-nucleotide repeat motifs observed across all the studied genomes whereas there were four species which lacked tetra-nucleotide motifs (Supplementary file 3). The tract size of the microsatellites has been shown in Fig. 2 and their motif composition in Fig. 3 while the details are provided in Supplementary file 2 and Supplementary file 3 respectively.

The tract size of mono- to tetra nucleotide repeats revealed that the highest tract size being contributed by tri-nucleotide repeats was observed in 11 species followed by di-nucleotide repeats in 9 species and mono-nucleotide repeats in 2 species. Individually, HEV22 had the highest tract size of 128 bases from di-nucleotide repeats followed by HEV19 with 114 from tri-nucleotide repeats.

The prevalent motif composition of mono- to tri-nucleotide repeats have been shown in Figure 3 and reflected the GC rich genome composition of studied viruses. The most prevalent mono-nucleotide repeat was “C” comprising of around 62% (69 of 112) of the mono-nucleotide SSRs. The other three motifs herein were almost equally represented with G(14), A(16), and T(13) incidences. Similarly, for di- and tri-nucleotide repeats the most prevalent motif was GT/TG and GAC/CTG respectively.

Thereafter, we investigated the possible correlation of genome size and GC content on number of SSR and cSSR incidence, RA, RD, of SSRs and cSSRs and cSSR% in SSR. GC content of assessed HEV genomes had a positive and strong correlation on cSSR incidence (*r* = 0.290267; *p* < 0.009679), cRA (*r* = 0.283470; *p* < 0.010746), cRD (*r* = 0.214411; *p* < 0.029992), and cSSR% (*r* = 0.294344; *p* < 0.009087) but GC content showed non-significant correlation on SSR incidence (*r* = 0.001397; *p* = 0.868796), sRA (*r* = 0.000290; *p* = 0.939940), and sRD (*r* = 0.009104; *p* = 0.672734). On the other hand, genome size show non-significant correlation of SSR (*r* = 0.008163; *p* = 0.689248), cSSR (*r* = 0.016400; *p* = 0.570061), sRA (*r* = 0.003596; *p* = 0.790937), cRA (*r* = 0.010717; *p* = 0.646612), sRD (*r* = 0.000092; *p* = 0.966178), cRD (*r* = 0.003083; *p* = 0.806111), and cSSR% (*r* = 0.053724; *p* = 0.299303).

### Microsatellite distribution

**Fig. 4:**
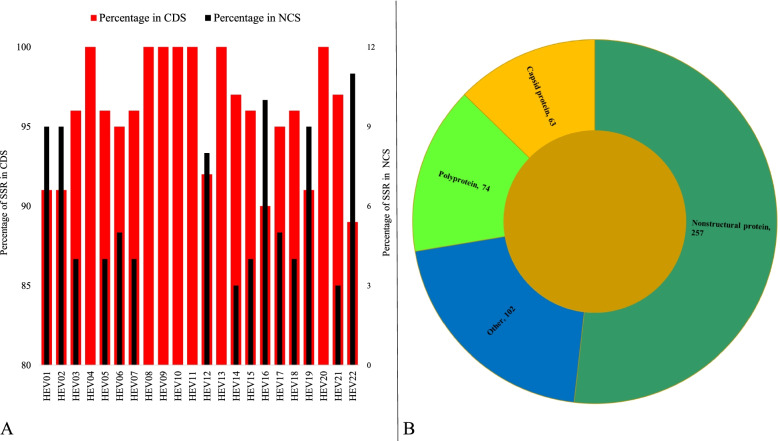
Distribution of SSRs in Hepeviridae genomes. **A** Coding and non-coding regions. **B** Different proteins

The location of incident SSRs across genomes has been represented in Fig. 4 and details in Supplementary file 3. All the studied genomes had a minimum of ~90% incident SSRs present in the coding regions and ~ 3% in non-coding regions of the genome (Fig. 4A). Seven species HEV03, HEV8, HEV9, HEV10, HEV11, HEV13, and HEV20 had SSRs localized exclusively to the coding region of the studied genomes. The protein-specific localization revealed almost 50% of the SSRs (257) to be present in non-structural protein with polyprotein coming in a distant second position with 74 SSRs (Fig. 4B).

**Fig. 5:**
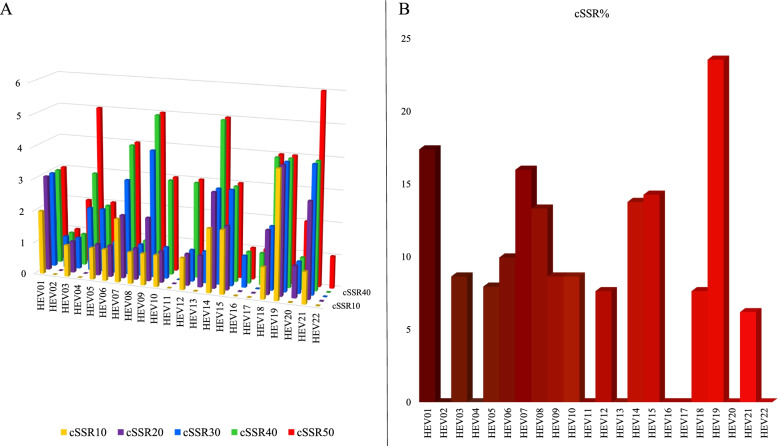
Clustering of SSRs in Hepeviridae genomes. **A** cSSR%. **B** cSSR incidence with varying dMAX (10–50)

Another approach to assess the distribution of SSRs is to analyze the cSSRs which in turn is a reflection on the clustering of SSRs. This is accomplished by analyzing the cSSR incidence by varying dMAX. cSSR incidence has been discussed before for dMAX of 10. The values of dMAX can be varied from 10 to 50 in IMEx and the corresponding cSSR incidences has been represented in Fig. 5A with details mentioned in Supplementary file 4. The increase in cSSR incidence is expected with increasing dMAX and was observed herein as well. However, the rate of increase is neither uniform nor follows any single priority rule.

The second aspect about cSSRs worth elaborating is cSSR% which represents the fraction of SSRs as part of cSSR. A higher cSSR% would represent regions of the genome with clustering of SSRs which can therein serve as hotspots of genome evolution. The observed cSSR% for dMAX of 10 has been shown in Fig. 5B and details mentioned in Supplementary file 1. cSSR% ranged from 0 (8 genomes with no cSSR) to 23.53% (HEV19). Thus, the uniqueness of the genome SSR signature is reiterated not only in terms of incidence and composition as discussed before but also in terms of localization and clustering.

### Phylogenetic analysis

**Fig. 6:**
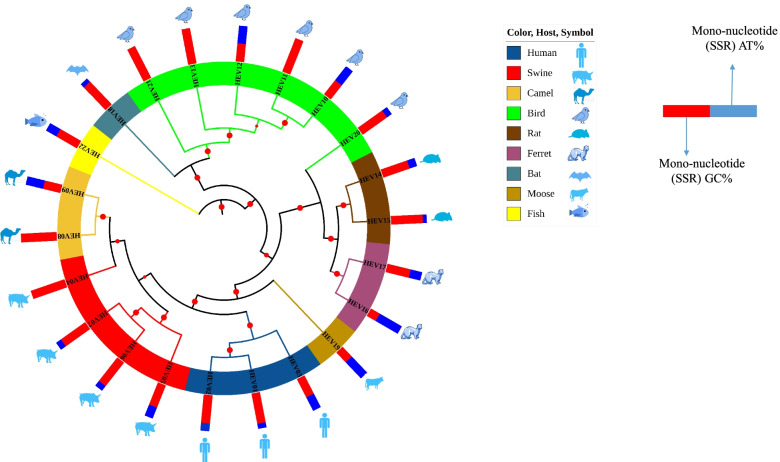
Phylogenetic analysis of the HEV genomes and mono-nucleotide SSR AT%

The only way to ascertain the role of SSRs in the evolution of viruses is to perform the phylogenetic analysis of genomes in context of some specific aspect. The phylogenetic tree for the studied genomes has been shown in Fig. 6. The host is one of the defining aspects of any virus and as evidently in the figure, viruses with same or similar hosts are placed together on the tree implicating viral host being one of the driving forces for evolution.

## Discussion

An increase in SSR incidence if accompanied by an increased cSSR would have suggested an incidence pattern for the microsatellites. However, non-conformation to any pattern is indicative of genome-specific SSR signature which is crucial for its evolution. The variation in SSR incidence has multiple facets. Not only are there genomes with 28 incident SSRs but no cSSR (HEV4 and HEV22) there is also HEV15 with 2 cSSRs for the same number of SSRs. Further the other three genomes with two cSSRs: HEV1, HEV7 and HEV14 have 23, 25, and 29 SSRs respectively. Thus, the SSRs are neither conforming to any rule in occurrence nor in clustering across studied genomes. We subsequently analyzed the diversity in motif composition and tract size of the incident microsatellites.

In terms of tract size, if we compare species with same number of incident SSRs, the results are all the more intriguing. HEV1, HEV3, HEV9, and HEV10 have same number of 23 SSRs present in their genomes (Fig. 1, Supplementary file 1). However, if we look at their respective tract size distribution its highly variant. HEV1, HEV3, and HEV10 have the maximum tract size from di-nucleotide repeats with 77 bases (11 SSRs), 72 bases (12 SSRs), and 101 bases (16 SSRs) respectively whereas HEV9 has maximum tract size of tri-nucleotide repeats with 85 bases. Contrastingly, all the above four genomes have very low representation in terms of incidence as well as repeat size for mono-nucleotide repeats.

The diversity in incidence and composition of microsatellites has been observed across viruses with varying genome size, type, and GC content [11–14]. Thus, these aspects represent the SSR signature for each genome in an unique manner. Their role in genome evolution, regulation of gene expression and biomarkers for speciation has been elucidated but not yet fully understood. The presence and variations of SSRs in HEV genomes warrants for further analysis so we looked at their distribution across coding and non-coding regions in the genomes.

The presence of SSRs predominantly in the coding region of HEV genomes illustrated two important aspects. First, since the viral genome is mostly coding so SSRs will also be expectedly mostly present in protein coding areas of the genome. Secondly, since the SSRs are associated with gene expression and viral evolution, their localization in proteins becomes a pre-requisite which is validated by the data.

The phylogenetic tree was assessed with possible correlation between host and localization of mono-nucleotide repeats to specific genome regions. Previously, we have reported the existence of mono-nucleotide repeats exclusively to the A/T region of the genome for human and related hosts [15]. The same has also been observed in *Rhabdoviridae* and *Coronaviridae* (data not shown). The present data set doesn’t conform to the said pattern wherein three species with birds as host exhibited exclusivity of mono-nucleotide SSRs to G/C region of the genome. This may be attributed to the higher GC content of the presently studied genomes. This indicates that host range is definitely decided by multiple factors and genomes of varied compositions need to be analyzed to forge a widely acceptable rule.

## Conclusions

The HEV genomes have unique SSR signature with no observable pattern in incidence, composition or localization. However, these variations are the key to their evolution as indicated by clustering of species with similar hosts on the phylogenetic tree. The SSR map for all viral genomes is the key to understanding and predicting viral evolution.

## Methods

### Genome sequences

The complete genome sequences of 22 HEV genomes of the family *Hepeviridae* and genus *Orthohepevirus* (21 species) and *Piscihepevirus* (1 species) were extracted from NCBI database (http://www.ncbi.nlm.nih.gov/). A summary of the studied species (genome type, classification, abbreviations, host) and genome features (size, GC%) have been mentioned in Supplementary file 1. The hosts information was extracted from Virus-Host Database from the given link (https://www.genome.jp/virushostdb/note.html).

### Microsatellite extraction and analysis

The extraction of microsatellites was done using Imperfect Microsatellite Extractor (IMEx) which yields mono- to hexa-nucleotide repeat motifs. The ‘Advance-Mode’ of IMEx with the previously established parameters was used. Minimum repeat size allowed was 6 (mono-), 3 (di-), 3 (tri-), 3 (tetra-), 3 (penta-), and 3 (hexa-) [16, 17].

Imperfect microsatellites were allowed and compound microsatellites (cSSR) defined by two or more SSRs separated by a distance of ≤ dMAX were extracted at varying dMAX from 10 to 50 at intervals of 10. Other parameters were set to the defaults. The extracted microsatellites were saved in Microsoft Office Excel 2019 and used for further analysis which included relative abundance (RA) and relative density (RD) for SSRs and cSSRs (sRA and sRD/cRA and cRD respectively); cSSR%; motif composition; tract size; localization on the genome and others. RA is defined as the number of microsatellites present per kb of the genome whereas RD is the number of bases present as SSR per kb of the genome. cSSR% represents the number of SSRs present as a part of cSSR expressed as percentage.

### Phylogenetic analysis

Alignment and phylogenetic reconstructions were performed using the function “build” of ETE3 v3.1.1 [18] as implemented on the GenomeNet (https://www.genome.jp/tools/ete/). Alignment was performed with MAFFT v6.861b with the default options [19]. The resulting alignment was cleaned using the gappyout algorithm of trimAl v1.4.rev6 [20]. Best nucleotide model was selected using ML tree inference among JC, K80, TrNef, TPM1, TPM2, TPM3,TIM1ef, TIM2ef, TIM3ef, TVMef, SYM,F81, HKY, TrN, TPM1uf, TPM2uf, TPM3uf,TIM1, TIM2, TIM3, TVM and GTR models using pmodeltest v1.4. ML tree was inferred using RAxML v8.1.20 ran with model GTRGAMMA and default parameters [21]. Branch supports were computed out of 100 bootstrapped trees.

### Correlation analysis

Correlation analysis was conducted to determine if genome size and GC content impact SSR incidence features. The simple correlation calculations were performed using Microsoft Office Excel 2016.

## Supplementary Information

**Additional file 1: Supplementary file 1.** Genomes features and extracted microsatellites of HEV in the study

**Additional file 2: Supplementary file 2.** SSR incidence, tract size, composition and location in HEV genomes

**Additional file 3: Supplementary file 3.** SSRs extracted from HEV genomes

**Additional file 4: Supplementary file 4.** cSSRs and varying dMAX in HEV genomes

## Data Availability

All data generated or analyzed during this study are included in this published article [and its supplementary information files] Studies involving plants must include a statement specifying the local, national or international guidelines and legislation and the required or appropriate permissions and/or licences for the study. Not applicable.

## Abbreviations

cSSR: Compound SSR
HEV: Hepatitis E virus
IMEx: Imperfect microsatellite extractor
RA: Relative abundance
RD: Relative density
SSR: Simple sequence repeats
